# Effect of Scrap Configuration on the Tensile and Flexural Properties of Grinding-Free Recycled CF/PA6 Composites

**DOI:** 10.1021/acsomega.6c08579

**Published:** 2026-10-14

**Authors:** Talita Melo do Nascimento, Larissa Stieven Montagna, Thiely Ferreira da Silva, Rieyssa Maria de Almeida Corrêa, Guilherme Ferreira de Melo Morgado, Juliano Marini, Camila Alves Escanio, Mirabel Cerqueira Rezende, Fabio Roberto Passador

**Affiliations:** † 28105 Federal University of São Paulo (UNIFESP), Polymer and Biopolymer Technology Laboratory (TecPBio), 330 Talim St., São José dos Campos 12231-280, Brazil; ‡ 67828 Federal University of São Carlos (UFSCar), Department of Materials Engineering, Rod. Washington Luiz, Km 235, São Carlos 13565-905, Brazil; § National Institute for Space Research (INPE), 54540 Coordination of Applied Research and Technological Development (COPDT), 1758 Astronautas Avenue, São José dos Campos 12.227-010, Brazil

## Abstract

The increasing demand for lightweight, high-performance materials in the automotive sector has driven the use of carbon fiber/polyamide 6 (CF/PA6) composites. This study proposes a grinding-free recycling route based on the reassembly of CF/PA6 composite cutouts into a controlled laminate configuration, followed by hot compression molding. Three configurations were investigated: S21 (two layers of 1 mm-thickness square cutouts), S13 (one layer of 3 mm-thickness square cutouts), and R21 (two layers of 1 mm-thickness rectangular cutouts), all containing 85 wt % CF/PA6 scraps and 15 wt % neat PA6 powder. The recycled laminates were characterized by ultrasonic inspection, differential scanning calorimetry (DSC), acid digestion for volumetric fraction determination, tensile and flexural testing, and field-emission gun scanning electron microscopy (FEG-SEM) analyses. Ultrasonic inspection and acid digestion showed that the S13 configuration exhibited the highest structural homogeneity and the lowest void content. In contrast, S21 and R21 presented higher void fractions, as confirmed by ultrasonic maps, acid digestion results, and FEG-SEM observations. Despite the higher void content, S21 achieved the highest tensile strength, probably due to the more efficient stress distribution throughout the laminate. Fractographic analyses revealed good fiber–matrix interfacial adhesion, as well as typical aspects observed in the fracture surfaces of thermoplastic polymeric composites, including plastic deformation, fiber pullout, and matrix-rich regions. Overall, the results demonstrate that the proposed grinding-free recycling approach enables the reuse of CF/PA6 waste in multilayer architectures in applications that do not require high structural performance.

## Graphical abstract

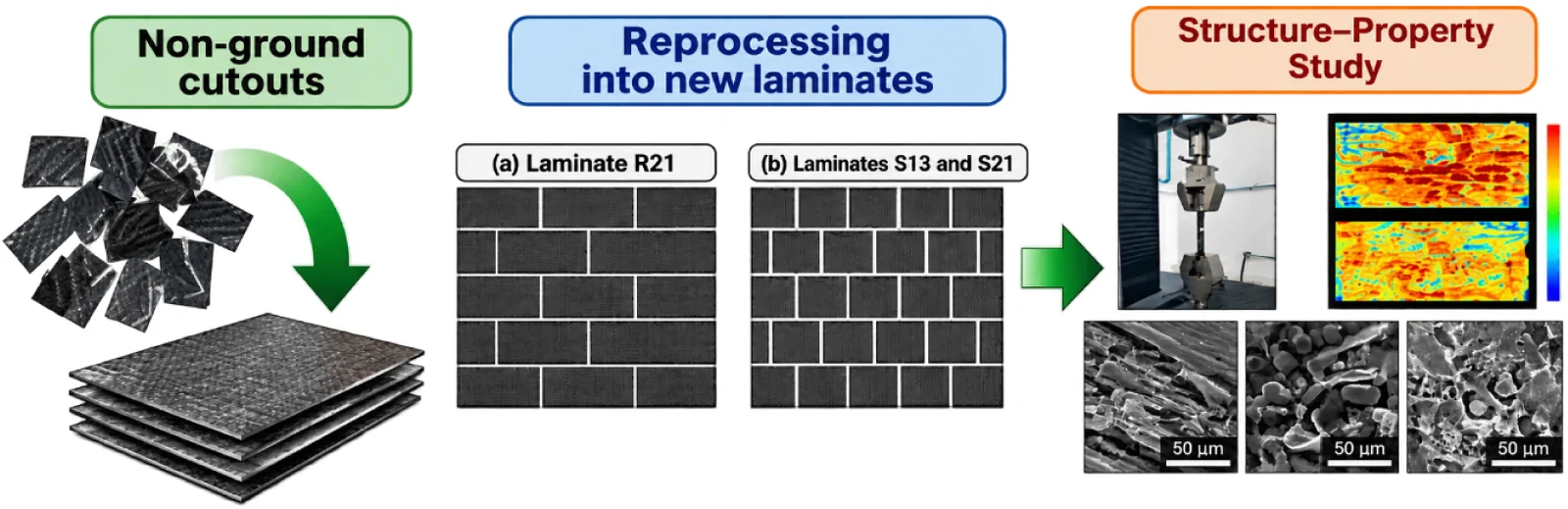

## 1 Introduction

Carbon fiber-reinforced polymer composites (CFRPs) are widely recognized as advanced structural materials due to their high specific strength, low density, and durability, which enable their extensive use in aerospace, automotive, wind energy, and civil engineering applications.^1^ In the mobility sector, CFRPs play a key role in lightweight design strategies, contributing to improved energy efficiency and reduced emissions.^2^ However, the rapid expansion of CFRP production has led to a significant increase in both pre- and postconsumer waste, raising critical challenges for sustainable materials management.^3,4^

Thermoplastic-based composites, particularly those reinforced with carbon fiber and polyamide 6 (CF/PA6), have gained increasing attention due to their combination of attractive mechanical performance and reprocessability.^5–7^ Unlike thermoset systems, thermoplastic matrices enable recycling through remelting-based processes such as hot compression molding, offering a promising pathway for circularity.^2^ Despite this potential, large-scale recycling of CFRPs remains limited by technical and economic constraints, resulting in the disposal of high-value materials that still retain significant structural integrity.^8,9^

Conventional CFRP recycling routes include mechanical, thermal, chemical, and electrochemical processes.^10,11^ Among these, mechanical recycling is the most industrially accessible; however, it usually involves grinding, which severely reduces fiber length and compromises mechanical performance.^12^ Alternative approaches based on thermal and chemical processes can partially recover fiber properties but are often associated with high energy consumption and environmental concerns.^12^ As a result, there is growing interest in recycling strategies capable of preserving fiber architecture while maintaining process simplicity and sustainability.

Compression molding has been investigated as a strategy for incorporating carbon-fiber composite waste into new components while retaining discontinuous reinforcement at larger length scales. Stelzer et al.,^13^ for example, investigated multilayered hybrid CF-SMC structures incorporating secondary carbon-fiber materials and demonstrated the potential of controlling material architecture to maintain manufacturability and structural performance. More recently, Santos et al.^14^ proposed a grinding-free mechanical recycling route specifically for CF/PA6 production waste, in which composite cutouts were randomly arranged in an aluminum mold and combined with different contents of neat PA6 (10, 20, 30, and 40 wt %) before hot compression molding. The resulting laminates showed improved consolidation with increasing PA6 content and promising mechanical performance. However, the influence of a systematically controlled scrap architecture, particularly cutout geometry and stacking configuration, remains insufficiently explored. This aspect provides the basis for the present study, in which the composition is maintained constant while the architecture of the CF/PA6 cutouts is varied.

Montagna et al.^15^ also employed a nongrinding recycling approach in which geometric scrap cutouts were arranged in a single layer and consolidated using PA6 pellets. The authors incorporated 40 wt % of virgin PA6 as the matrix phase and demonstrated that the geometry and dimensions of the cutouts markedly affect the mechanical behavior, with larger fiber segments promoting superior mechanical performance.

In this context, this study proposes a grinding-free recycling strategy for CF/PA6 composites based on scrap reprocessing and controlled reassembly. Unlike previous approaches relying on random scrap distribution,^14,15^ the present work investigates the effect of predefined geometric arrangements of cutouts (squares and rectangles), organized in distinct and multilayered configurations, using 85 wt % of composite cutouts and 15 wt % of neat PA6 in all laminates, on the resulting laminate properties. The composition investigated in this study was selected considering the primary objective of maximizing the incorporation of postproduction CF/PA6 composite waste while limiting the amount of additional virgin polymer required for reprocessing. Although a higher PA6 content (40 wt %) was previously reported to provide improved mechanical performance,^14^ directly adopting this composition would substantially decrease the recycled-material fraction of the resulting laminate. Therefore, a formulation containing 85 wt % recycled CF/PA6 composite cutouts and 15 wt % additional PA6 was selected as a compromise between recycled-material content and processing requirements. The additional PA6 was incorporated to facilitate polymer redistribution during hot compression molding, improve contact and bonding between adjacent composite fragments, and promote laminate consolidation, while maintaining the recycled composite as the predominant constituent. Thus, unlike the optimization criterion adopted by Santos et al.,^14^ which emphasized mechanical performance, the composition used here was selected to balance high waste incorporation, limited virgin-material consumption, processability, laminate consolidation, and mechanical performance. The recycled materials are systematically characterized through ultrasonic inspection, differential scanning calorimetry (DSC), fiber volume fraction measurements, tensile and flexural testing, and field emission gun scanning electron microscopy (FEG-SEM).

By establishing relationships between scrap architecture and mechanical performance, this work contributes to the development of more efficient and scalable recycling strategies for thermoplastic composites, supporting the transition toward circularity in high-performance materials.

## 2 Experimental Section

### 2.1 Materials

Postprocessing residues derived from a carbon fiber-reinforced polyamide 6 (CF/PA6) composite, commercially designated by Toray as Cetex TC910PA6 T700SC-12k, were employed in this study. The material was supplied in two different forms: a) semipregs with 1 mm-thickness and b) a laminate with 5 plies and 3 mm-thickness. Both materials contain approximately 39 wt % PA6 content and the same CF arrangement—namely, 2 × 2 twill fabric (415 g·m^–2^, 12k tow), according to the supplier (Toray Advanced Composites/Netherlands).

The PA6 used as matrix was a commercial grade (Aegis H8202NLB), provided by AdvanSix (USA). This material exhibits a density of 1.13 g/cm^3^ and a melt flow index (MFI) of 9.8 g/10 min (235 °C, 1 kg), supplied in pellet form.

### 2.2 Recycling Process

A grinding-free recycling approach was adopted for the CF/PA6 composite waste to preserve fiber length and to minimize structural damage. The composite residues were sectioned into smaller pieces using heavy-duty metal shears, due to the high stiffness and cutting resistance of the material, yielding controlled geometries of 25 mm × 25 mm (squares, named as S) and 50 mm × 25 mm (rectangles, named as R). The CF/PA6 semipreg was cut into squares (code S) and rectangles (code R), and the CF/PA6 laminate into squares (S). Surface irregularities and edge defects were subsequently removed by sanding to ensure uniformity.

In contrast, the neat PA6 was used in powder form as a polymeric matrix. PA6 pellets were cryogenically ground using liquid nitrogen in an analytical mill (Ika A11, Ika), operating at 60 Hz and a maximum rotational speed of 28,000 rpm, equipped with an A11.2 cutting blade. The proportion of 85 wt % of composite cutouts and 15 wt % of neat PA6 was used in all laminates.

Before consolidation, both the CF/PA6 cutouts and the PA6 powder were dried in a laboratory oven (Solab SL-100, Solab) at 60 °C for 24 h to remove residual moisture. Figure 1 schematically illustrates the overall processing route, from waste preparation to specimen machining.

**Figure 1:**
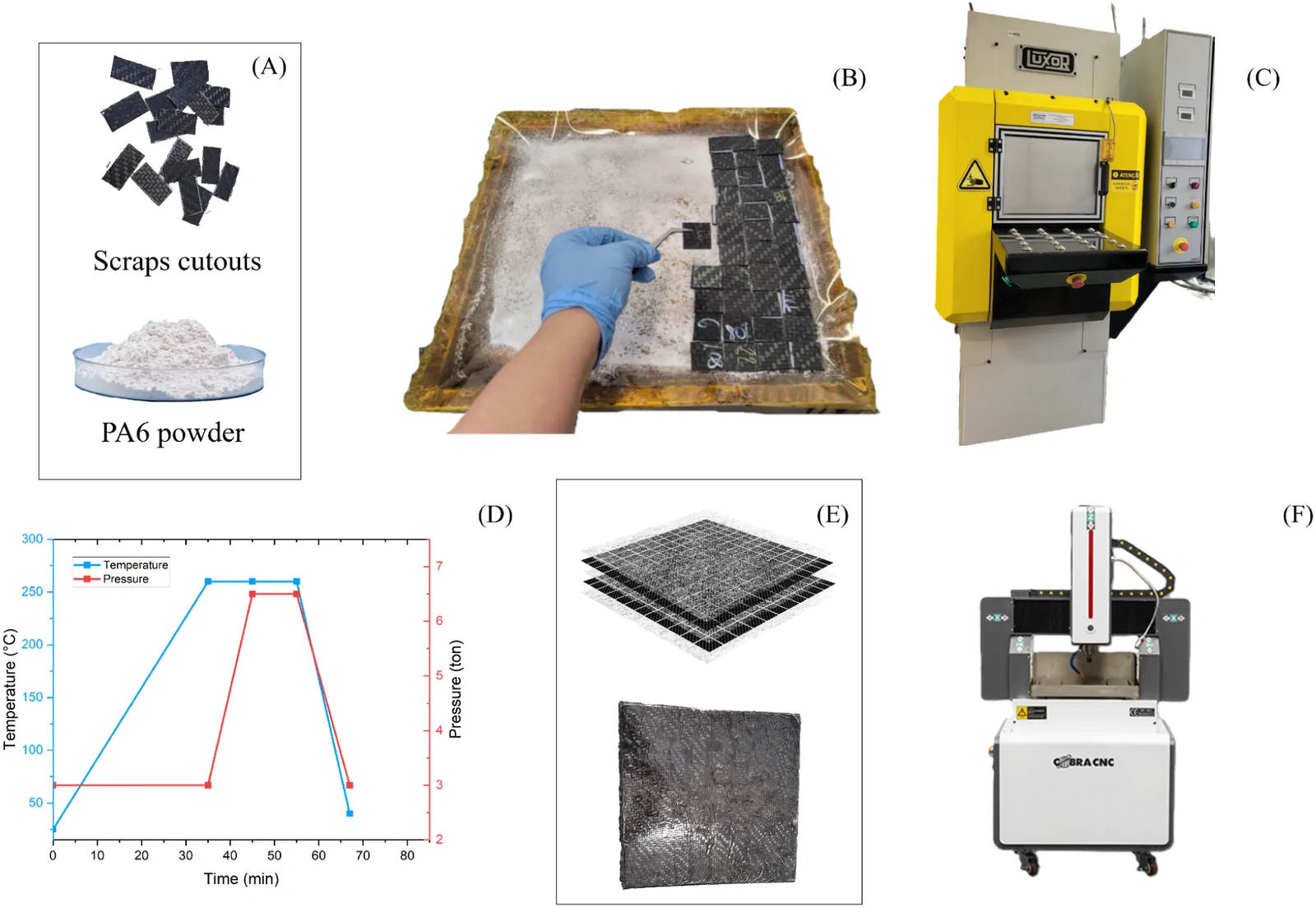
Schematic representation of the processing methodology: (A) CF/PA6 waste cutouts and neat PA6 powder; (B) layer assembly within an aluminum mold lined with polyimide film; (C) used hot-press equipment; (D) processing cycle used in the hot compression molding; (E) schematic of layer stacking and the resulting consolidated laminate; and (F) CNC machining of laminates into standardized test specimens.

For obtaining the laminates, an initial layer of neat PA6 powder was deposited in the aluminum mold lined with polyimide film, followed by the arrangement of CF/PA6 cutouts in a butt-joint configuration, without overlapping the adjacent pieces (cutouts). The layers were assembled by placing the cutouts side by side, with the cutouts in the subsequent row positioned with an offset, similar to the arrangement seen in a brick wall (Figure 2). Subsequent layers were built by alternately adding neat PA6 powder between the composite cutout layers, with a final top layer of PA6 to complete the stacking sequence. The total mass of PA6 (15 wt %) was equally divided among the laminate layers.

**Figure 2:**
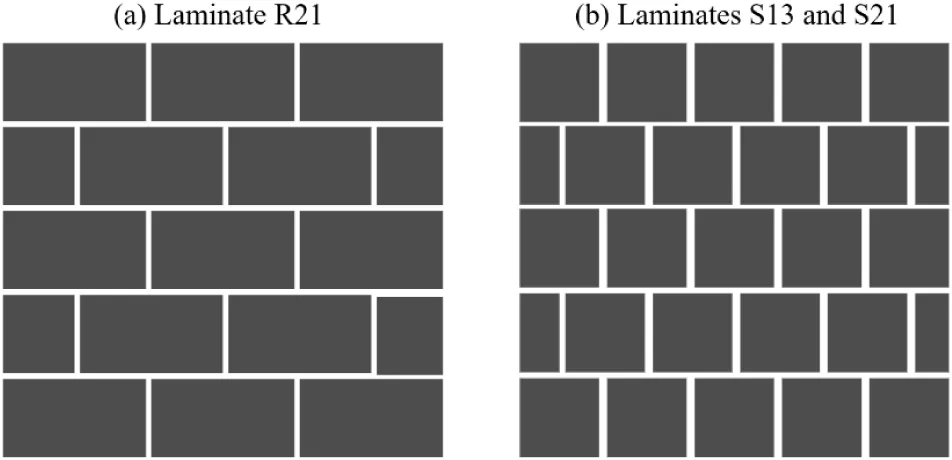
Representation of laminate configurations showing the stacking sequence. (a) R21 (two layers semipreg rectangular cutouts with 1 mm-thickness), (b) S21 (two layers semipreg rectangular cutouts with 1 mm-thickness), and S13 (one layer laminate square cutouts with 3 mm-thickness).

The stacked assembly was consolidated via hot compression molding using a Luxor press (Lab 40.15/1/Era.Aut-of 4272). The material, arranged in the mold at room temperature, was inserted into the press and subjected to a heating rate of 7 °C·min^–1^ under an initial pressure of 3 t until reaching 260 °C. The mold was held at this temperature for 20 min. The pressure was then increased to 6.5 t and held for an additional 20 min to promote consolidation. The used processing cycle is shown in Figure 1D.

Subsequently, the mold was cooled at a controlled rate of 20 °C·min^–1^ down to 40 °C. After removal from the press, the mold remained at ambient conditions for 30 min before demolding, allowing gradual cooling to room temperature. This procedure minimizes the laminate warpage. This processing route was adapted from the methodology reported by Santos and collaborators.^14^ Figure 2 summarizes the laminate configurations, including stacking sequence and geometric forms.

### 2.3 Recycled Laminates Characterization

#### 2.3.1 Ultrasound Analysis

A nondestructive ultrasonic test was carried out to evaluate the internal quality of the consolidated laminates using an OmniScan X3 system (Olympus Corporation), operated with OmniScan MXU 5.18.1 software. A phased array configuration was employed, utilizing a 3.5 MHz transducer with 64 elements. This enabled the conversion of attenuation signals into high-resolution images and the generation of detailed ultrasonic maps of the inspected regions.

#### 2.3.2 Volumetric Fraction by Acid Digestion

The volumetric fractions of fiber, matrix, and voids were determined according to ASTM D3171. The method is based on selective matrix removal via acid digestion while preserving the carbon fibers. All tests were performed in triplicate.

Figure 3 illustrates the acid digestion procedure used in this work. Samples (15 mm × 15 mm) were dried at 60 °C for 24 h, weighed, and immersed in 20 mL of concentrated sulfuric acid (H_2_SO_4_, Neon, Brazil). The solution was heated to 150 °C on a hot plate (Corning PC-420D) for 4 h to promote matrix digestion. Subsequently, 5 mL of hydrogen peroxide (H_2_O_2_, 30% v/v, Anidrol, Brazil) was added to complete the digestion process and promote the flotation of the carbon fibers in the solution. Then, the fibers were washed three times with distilled water and dried at 105 °C for 24 h. After cooling in a desiccator for 1 h, the fibers were weighed.

**Figure 3:**
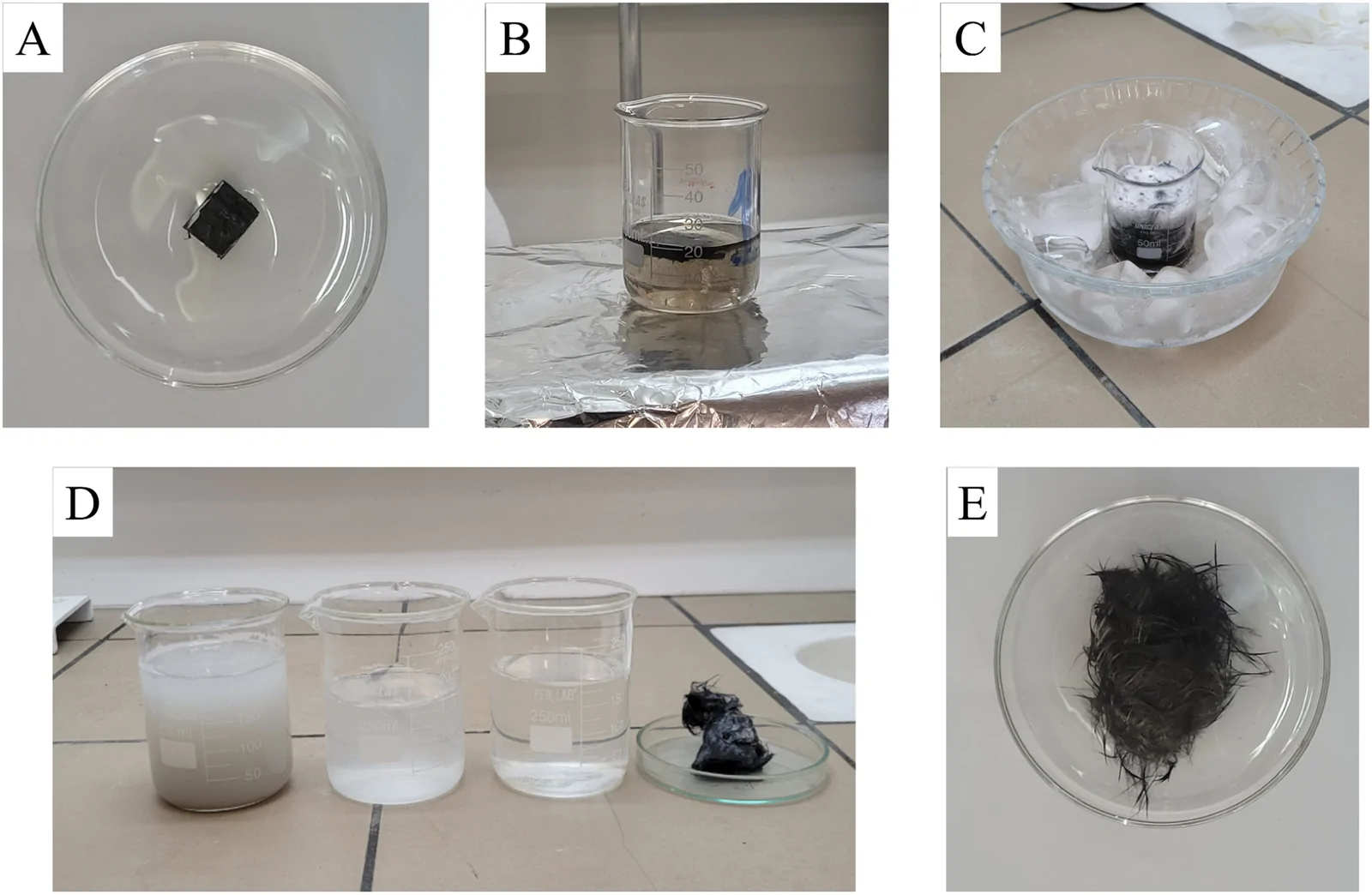
Main steps of the acid digestion of the samples: (A) sample; (B) heating in sulfuric acid; (C) ice bath with sulfuric acid and hydrogen peroxide; (D) three-stage washing with distilled water and wet fibers; (E) dried carbon fibers.

Using the measured values, the reinforcement volume fraction (V_r_), matrix volume fraction (V_m_), and void volume fraction (V_v_) were calculated, according to eqs 1, 2, and 3.

1 $$V_{r}=\left(\frac{w_{f}}{w_{i}}\right)\times \left(\frac{\rho_{c}}{\rho_{r}}\right)\times 100$$

2 $$V_{m}=\left(\frac{w_{i}-w_{f}}{w_{i}}\right)\times \left(\frac{\rho_{c}}{\rho_{m}}\right)\times 100$$

3 $$V_{v}=100-\left(V_{r}+V_{m}\right)$$

Where, composite density (ρ_*c*_) was determined from the specimen mass-to-volume ratio, while the fiber density (ρ_*r*_) was considered as 1.76 g·cm^–3^ and the PA6 matrix density (ρ_*m*_) as 1.14 g·cm^–3^.^16^ The values of *w*_*i*_ (initial composite mass before digestion) and *w*_*f*_ (fiber mass after digestion) were obtained by weighing the composite and the recovered fibers, respectively.

#### 2.3.3 Thermal Analyses

The thermal behavior of neat PA6, CF/PA6 composite waste, and recycled CF/PA6 laminates was evaluated by DSC, using a TA Instruments Q2000 equipment. Nitrogen was used as the purge gas at a flow rate of 50 mL·min^–1^. The thermal program consisted of: (i) heating from −20 to 300 °C at 10 °C·min^–1^; (ii) an isothermal step at 300 °C for 3 min to erase the thermal history; (iii) cooling from 300 °C to −20 °C at 10 °C·min^–1^; and (iv) a second heating from −20 to 300 °C at 10 °C·min^–1^. Equation 4 was used to calculate the degree of crystallinity (*X*_*c*_) of PA6 from the melting enthalpy obtained by DSC.

4 $$X_{c}=\frac{\Delta H_{m}}{w_{m}\times \Delta H_{m}^{0}}\times 100$$

where: *ΔH*_*m*_ is the melting enthalpy obtained by DSC, *ΔH*_*m*_^0^ is the standard melting enthalpy of PA6 (*ΔH*_*m*_^0^ = 230 J/g for 100% crystalline PA6),^17^ and *w*_*m*_ is the mass fraction of PA6.

#### 2.3.4 Mechanical Properties

Tensile and flexural tests were conducted in accordance with ASTM D3039/D3039M-17 and ASTM D7264/D7264M-21, respectively, using a universal testing machine (MTS Criterion 45) equipped with a 50 kN load cell for both and a crosshead speed of 2 mm/min for the tensile test and 1 mm/min for the flexural testing. Five specimens per laminate were tested.

Standardized specimens were machined for tensile and flexural testing, according to the standards, being five specimens of 250 mm × 25 mm × 2–3 mm of thickness for the tensile tests and five of 84 mm × 25 mm × 2–3 mm of thickness for the flexural tests, for each laminate configuration. Cutting was performed using a Cobra CNC RC 4040 milling machine equipped with a 3 mm diameter multiflute cutter coated with titanium nitride. The test specimens of the R21 laminate, obtained using rectangular cutouts, were cut with their long axis aligned with the rectangle’s longer dimension. Consequently, in the tensile tests, these specimens featured longer fibers aligned with the direction of the applied load.

Statistical evaluation was performed using one-way analysis of variance (ANOVA), followed by Tukey’s honestly significant difference (HSD) test for multiple comparisons. Analyses were conducted using OriginPro 2025 software (OriginPro), considering results from tensile strength and flexural behavior.

#### 2.3.5 Fractographic Analysis

Fractographic analyses were performed using a FEG-SEM microscope (Tescan, model MIRA 3) operating at 5 keV. Fracture surfaces from selected tensile specimens were mounted on stubs and sputter-coated with gold using a Quorum Q150RS Plus for 120 s before observation.

## 3 Results and Discussion

### 3.1 Ultrasonic Inspection

Figure 4 presents the visual appearance and ultrasonic inspection of the recycled CF/PA6 laminates, highlighting the influence of cutout geometry and stacking configuration on consolidation quality and structural integrity. The ultrasonic maps provide further insight into the internal quality of the laminates through color-coded images, in which transmission levels range from 0% (white, indicating defects) to 100% (red, indicating full consolidation), as indicated by the scale bar on the right side of the image.

**Figure 4:**
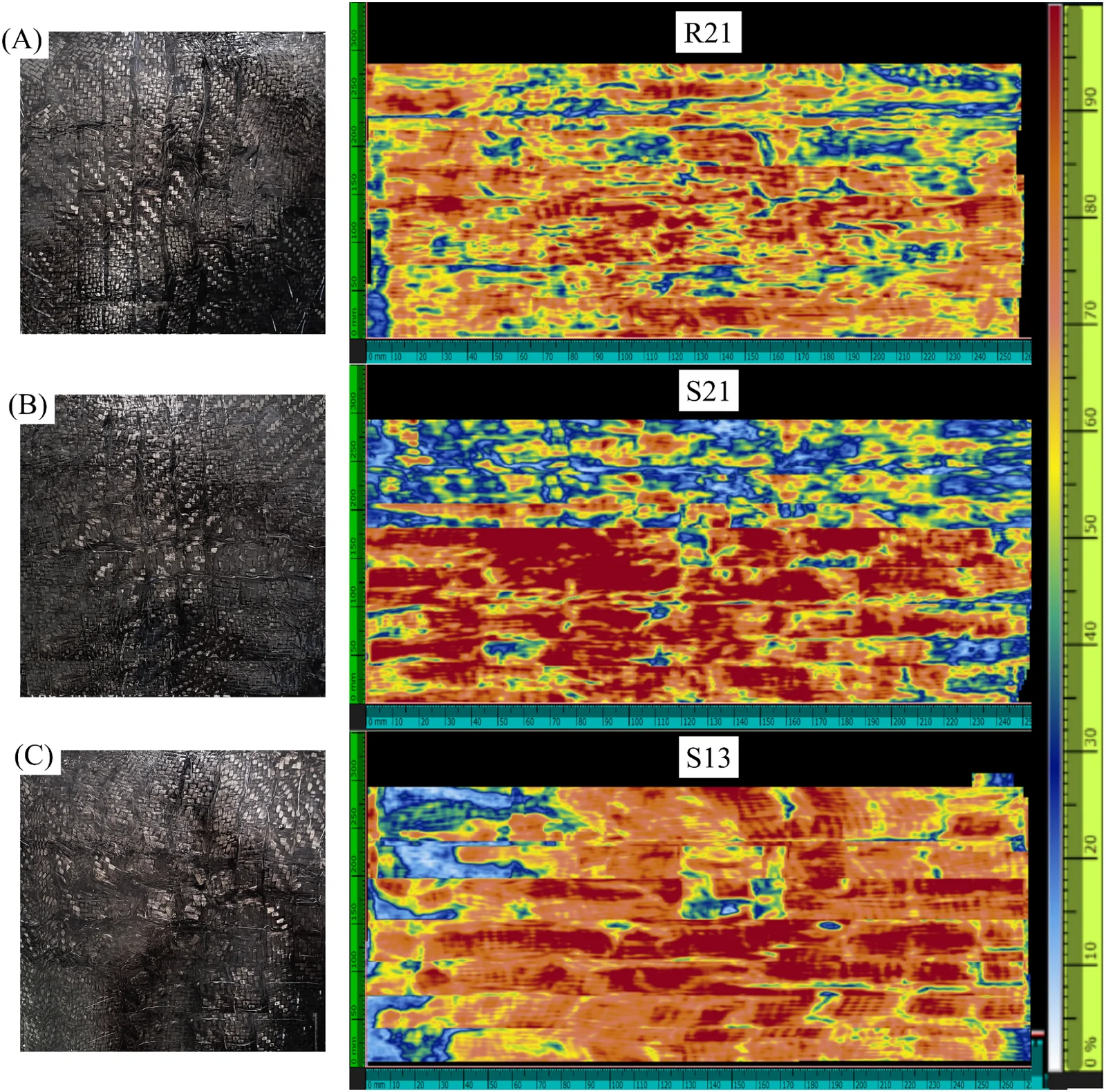
Visual appearance and ultrasonic inspection of the recycled CF/PA6 laminates: (A) R21 laminate with two layers of rectangular cutouts with 1 mm-thickness (50 mm × 25 mm); (B) S21 laminate with two layers of square cutouts with 1 mm-thickness (25 mm × 25 mm); and (C) S13 laminate with a single layer of square cutouts with 3 mm-thickness (25 mm × 25 mm).

Laminate R21, composed of rectangular cutouts of 1 mm-thickness (50 mm × 25 mm) arranged in a double-layer configuration, exhibits regions of both satisfactory and poor consolidation distributed throughout the laminated plate, suggesting slippage of the cutouts during the hot compression process. In contrast, laminate S21 (double-layer, 25 mm × 25 mm cutouts with 1 mm-thickness) showed a distinct consolidation profile, with poorly consolidated regions concentrated in the upper area, whereas the lower area exhibited improved consolidation, characterized by red regions corresponding to consolidation levels above 90%. Furthermore, the orthogonal fiber arrangement reduced the warpage observed in both laminates.

In contrast, the laminate S13 (25 mm × 25 mm) exhibited the most homogeneous ultrasonic response, with extensive red regions and only limited bluish areas near the left edges, indicating superior consolidation quality with fewer voids and defects. This behavior may be attributed to the good quality of the original laminated with five plies supplied by the Toray company, and also to the single-layer architecture, which reduced the available space for cutout slippage during the hot compression molding. Therefore, this laminate configuration minimized the void formation, since the cutouts used had already been previously consolidated. This observation is consistent with the visual inspection results, as the single-layer configuration restricted cutout displacement and promotes a more uniform matrix distribution, although a slightly higher degree of warpage was observed.

### 3.2 Volumetric Fraction by Acid Digestion

The volumetric fractions of matrix, fiber, and voids are presented in Table 1. The values of void content (*V*_*v*_), matrix content (*V*_*m*_), and fiber content (*V*_*f*_) were determined by the acid digestion method. Based on the obtained results, it is observed that the laminate S13 exhibited the highest fiber content (63.4%) and the lowest void fraction (1.5%), which may be attributed to its thicker cutouts (3 mm-thickness) containing a greater number of preconsolidated fiber layers. In contrast, the double-layer laminates (S21 and R21) presented intermediate fiber (∼31% and ∼53%, respectively) and matrix (∼48% and ∼34%, respectively) contents, accompanied by a larger void fraction (∼21% and ∼13%, respectively).

**Table 1:** Volumetric Fractions Obtained by Acid Digestion Analysis of the Recycled CF/PA6 Laminates[Table-fn tbl1fn1]

	**Volumetric fractions (%)**		
Samples	PA6	Carbon fiber	Voids
S13	35.1 ± 1.5^a^	63.4 ± 1.5^a^	1.5 ± 0.2^a^
S21	48.0 ± 1.7^b^	31.2 ± 0.2^b^	21.1 ± 1.5^b^
R21	33.9 ± 2.1^a^	53.2 ± 4.2^c^	13.1 ± 2.2^c^

i Different letters in the columns indicate significant differences according to ANOVA, at a Tukey significance level of 0.05.

In the case of the recycled laminate S21, a lower fiber percentage (∼31%) and a higher matrix percentage (48%) are observed compared to the other laminates. This behavior can be attributed to the greater heterogeneity in the consolidation regions, as observed for the laminate S21, as shown in Figure 4-B (ultrasound inspections). The fiber and matrix fraction results for this composite were possibly influenced by the fact that samples were taken from the upper region of the laminate, with more defects, as shown in Figure 4-B.

In principle, significant variations in fiber and matrix contents would not be expected, given that all laminates were produced using scraps with the same fiber and matrix content, as described in the Experimental Section. However, statistical analysis of the results confirms variations in fiber, matrix, and void contents, which are attributed to the heterogeneities observed in the recycled laminates, as observed in the ultrasonic inspections. Thus, a direct correlation was also observed between the acid digestion results and the ultrasonic inspection, indicating strong agreement between the two characterization techniques regarding the detection and distribution of voids within the laminates. Regions identified as poorly consolidated in the ultrasonic maps corresponded to laminates with higher void fractions measured by acid digestion, confirming the reliability and complementarity of both methods for assessing laminate quality.

Montagna et al.^15^ employed a similar nongrinding recycling strategy for CF/PA6 composites and reported average volumetric fractions of approximately 57% matrix, 38% fiber, and 3% voids. These values were associated with the incorporation of 40 wt % neat PA6 into the recycled laminates, which explains the relatively high matrix fraction observed in their study. In contrast, the present work incorporated only 15 wt % neat PA6; consequently, lower matrix contents were expected.

In summary, among the investigated configurations, the laminate S13, produced from a single layer of 3 mm-thickness square cutouts, exhibited the lowest void content and the most homogeneous consolidation profile. This behavior is likely associated with the thicker and more compact architecture of the cutouts, which already contained multiple preconsolidated fiber layers before the recycling processing. Such a configuration reduced interfacial gaps and restricted cutout displacement during hot compression molding, thereby minimizing void nucleation and growth. Furthermore, the single-layer arrangement promoted a more uniform stress and heat distribution throughout the laminate thickness, contributing to improved matrix flow and consolidation efficiency. This interpretation is fully supported by the ultrasonic inspection, which revealed predominantly red regions with minimal defect indications, confirming the superior structural integrity of the laminate S13.

### 3.3 Thermal Analysis

The thermal behavior of neat PA6, CF/PA6 composite scraps, and laminates S13, S21, and R21 was investigated by DSC, and the corresponding curves are presented in Figure 5. The first heating, cooling, and second heating cycles revealed the characteristic thermal transitions associated with PA6, including the glass transition, crystallization, and melting behavior.

**Figure 5:**
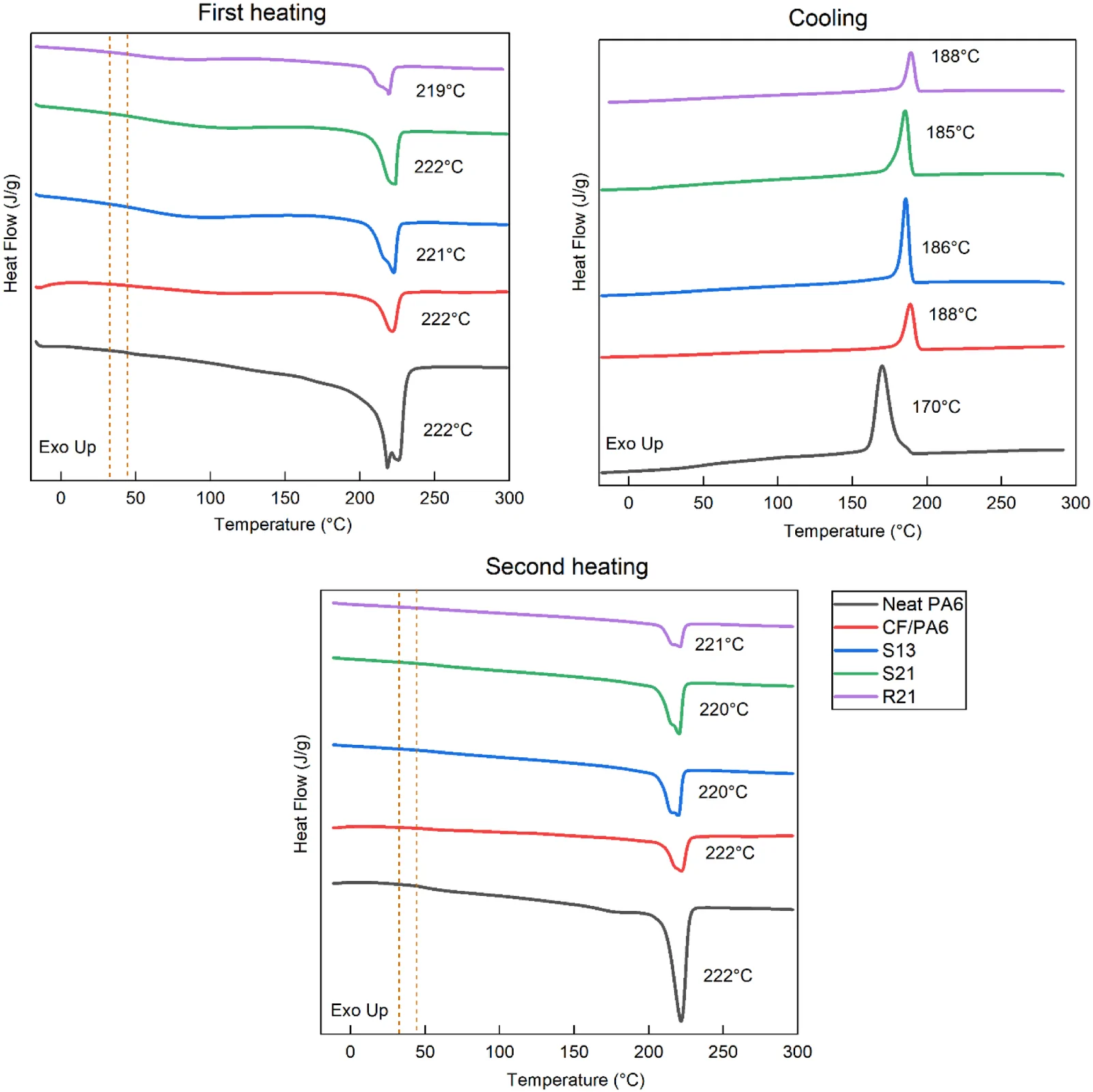
DSC curves of first heating, cooling, and second heating of neat PA6, CF/PA6 composite scraps, and laminates S13, S21, and R21. Dashed lines corresponding to *T*_g_ values region.

During the first heating cycle, the glass transition temperature (*T*_g_) varied from approximately 42 °C for the neat PA6 to 47 °C for the laminate S21. The slight increase in *T*_g_ observed for the recycled laminates may be associated with the constraining effect imposed by the carbon fibers on the mobility of the PA6 molecular chains, particularly in regions with higher fiber packing and improved interfacial interaction. The neat PA6 curve also exhibited two endothermic melting peaks accompanied by a shoulder in the melting region, a typical behavior of semicrystalline PA6. This feature is commonly attributed to the coexistence of different crystalline polymorphs, especially the α and γ phases, as well as melting–recrystallization phenomena occurring during the first heating cycle.^18^

In the second heating cycle, the *T*_g_ values remained within a similar range; however, the transition became less pronounced for the composite laminates. Additionally, the shoulder previously observed in the melting region disappeared after the controlled thermal cycle, indicating a reorganization of the crystalline structure during cooling from the melt. This suggests that the thermal history associated with the original processing of the laminates was erased during DSC analysis, resulting in the formation of a more homogeneous crystalline phase distribution upon recrystallization. The reduced prominence of *T*_g_ in the composites can also be explained by the lower fraction of amorphous polymer available for relaxation and by the restriction of chain segment mobility caused by the presence of carbon fibers and the preconsolidated composite architecture. Such effects are frequently reported in fiber-reinforced thermoplastic composites, in which the reinforcement phase limits molecular rearrangement and alters the relaxation dynamics of the polymer matrix.^19^

The melting temperature (*T*_m_) remained approximately constant near 222 °C for all samples, indicating that the crystalline structure of PA6 was not significantly altered by the presence of carbon fibers or by the recycling process. This result suggests that the reinforcement predominantly influenced the crystallization kinetics and morphology rather than the thermal stability of the crystalline phase itself.

During the cooling cycle, the crystallization temperature (*T*_c_) shifted from approximately 170 °C for the neat PA6 to nearly 188 °C for the composite laminates, demonstrating the strong nucleating effect of the carbon fibers. The presence of the fibers provided heterogeneous nucleation sites that facilitated earlier crystal formation and accelerated crystallization during cooling. Such behavior is commonly observed in fiber-reinforced thermoplastic composites and contributes to the development of a more refined and homogeneous crystalline morphology. Furthermore, the increase in *T*_c_ indicates enhanced crystallization kinetics in the recycled laminates, which may also be associated with residual oriented structures inherited from the preconsolidated CF/PA6 cutouts.

Table 2 summarizes the thermal parameters obtained from the cooling and second heating cycles of neat PA6, CF/PA6 composite scraps, and the recycled CF/PA6 laminates.

**Table 2:** Thermal Parameters from the Cooling, First and Second Heating of Neat PA6, CF/PA6 Scraps, and Recycled CF/PA6 Laminates

	**1°** **Heating**					**Cooling**	**2°** **Heating**			
Sample	*T*_g_ (°C)	*T*_m_ (°C)	ΔH_m_ (J/g)	*w*_m_ (%)	*X*_c_ (%)	*T*_c_ (°C)	*T*_g_ (°C)	*T*_m_ (°C)	ΔH_m_ (J/g)	*X*_c_ (%)
Neat PA6	45	222	61.4	1.0	26.7	170	44	222	52.0	22.6
CF/PA6	47	222	22.5	0.4	24.4	188	42	222	24.0	26.1
S13	45	221	28.5	0.4	31.0	186	46	220	23.2	25.2
S21	43	222	31.8	0.5	27.7	185	47	220	24.3	21.1
R21	46	219	14.0	0.4	15.2	188	44	221	11.3	12.3

The degree of crystallinity (*X*_c_) in the first heating was calculated based on the melting enthalpy values, resulting in 26.7% for the neat PA6 and approximately 24.4% for the PA6 phase in the CF/PA6 scraps. For the recycled laminates, the matrix weight fraction (*w*_*m*_), determined from the acid digestion results, was used to calculate the crystallinity of the polymer phase. Higher *X*_c_ values were obtained for the recycled laminates, reaching 31% for S13 and 27.7% for S21, in the first heating, even though the processing cycle was the same for all three laminates. When comparing these results with those obtained for neat PA6 (∼27%) and CF/PA6 scraps (∼25%), in the first heating, the influence of the reinforcement on polymer crystallization during the recycling process is observed.

The increase in crystallinity observed for the recycled laminates (S13 and S21) may be attributed to the thermal and processing history imposed during hot compression molding, which likely promoted molecular chain rearrangement and more efficient crystal organization within the PA6 matrix. In addition, the presence of carbon fibers can enhance heterogeneous nucleation during cooling, accelerating crystallization and favoring the formation of more ordered crystalline regions. The higher crystallinity observed for laminate S13 (31%) is also consistent with its superior consolidation quality and lower void content, as previously evidenced by ultrasonic inspection and acid digestion analyses.

In the case of laminate S21, although the upper region shows poor consolidation (bluish region in Figure 4), it exhibits a high degree of crystallinity (∼28%). This behavior can be attributed to the contribution of the lower region of the laminate, which is better compacted (reddish region in Figure 4). Conversely, laminate R21, which generally displays low consolidation across its entire area, showed inhibited growth of more ordered crystalline regions (∼15%). These *X*_c_ results show that improved consolidation may facilitate heat transfer and promote more uniform crystallization throughout the laminate thickness.

These results are in agreement with the data reported by Morgado et al.,^20^ reinforcing the interpretation that the reprocessing and the presence of carbon fibers significantly influence the crystallization behavior of PA6-based composites. Although the incorporation of fibers can restrict polymer chain mobility locally, the nucleating effect of the reinforcement and the thermal cycles associated with reprocessing appear to dominate the crystallization behavior in the recycled laminates.

Overall, the DSC results demonstrate that the recycled materials preserved the characteristic thermal behavior of PA6 (*T*_g_ and *T*_m_) after reprocessing, confirming the effectiveness of the proposed recycling strategy and supporting the potential recycling of CF/PA6 waste for the production of structurally consolidated thermoplastic composites.

### 3.4 Mechanical Properties

The stress–strain curves of the recycled laminates are presented in Figure 6, while the corresponding tensile properties are summarized in Table 3. Significant differences in mechanical performance were observed among the recycled configurations, highlighting the strong influence of cutout geometry, laminate architecture, and stacking sequence on the tensile behavior of the composites.

**Figure 6:**
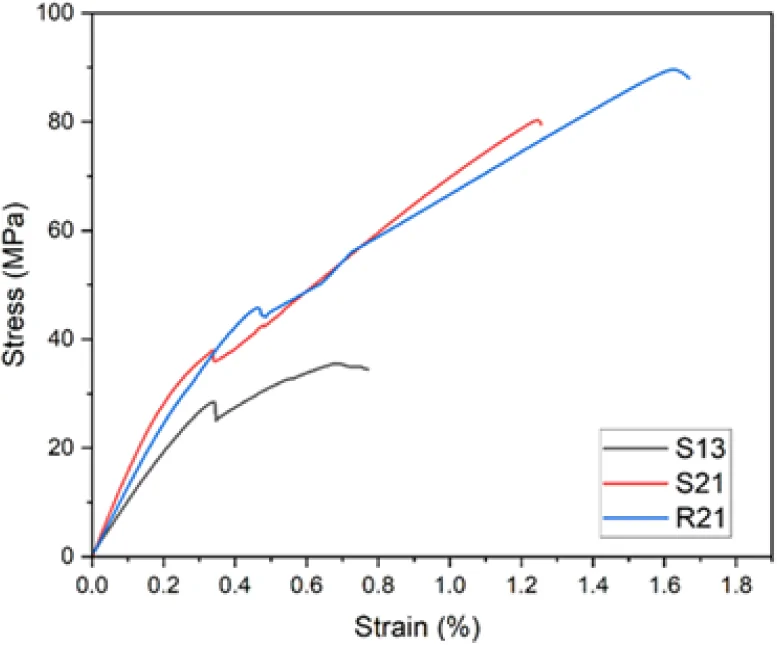
Mean stress–strain curves representing the mechanical behavior of recycled CF/PA6 laminates.

**Table 3:** Values of Ultimate Strength, Elastic Modulus, and Elongation at Break in Tensile and Flexural Tests for the Recycled CF/PA6 Laminates[Table-fn tbl3fn1]

	**Tensile test**			**Flexural test**		
Sample	Ultimate tensile strength (MPa)	Elastic Modulus (GPa)	Elongation at break (%)	Flexural strength (MPa)	Elastic Modulus (GPa)	Elongation at break (%)
S13	33.3 ± 1.6^a^	10.1 ± 2.0^a^	0.5 ± 0.2^a^	3.5 ± 1.0^a^	87.4 ± 23.7^a^	4.0 ± 0.4^a^
S21	62.5 ± 21.0^b^	16.2 ± 0.7^b^	1.3 ± 0.1^b^	3.0 ± 0.0^a^	79.7 ± 21.2^a^	4.0 ± 1.1^a^
R21	61.0 ± 24.8^b^	13.6 ± 1.7^b^	0.9 ± 0.6^b^	3.5 ± 0.6^a^	98.0 ± 7.3^a^	3.6 ± 0.8^a^

i Different letters in the columns indicate significant differences according to ANOVA, at a Tukey significance level of 0.05.

When comparing the tensile strength values measured for the processed thermoplastic composites in the present study with those reported by Morgado et al.^20^ for continuous-fiber CF/PA6 laminates, a significant decrease is observed, as expected. Morgado et al.,^20^ using the same CF/PA6 system, reported tensile strengths of 612 ± 35 MPa. Therefore, the recycled laminates retained between approximately 5% (S13) and 10% (S21 and R21) of the tensile strength of the continuous CF/PA6 laminate. The reduction in tensile strength compared with continuous-fiber laminates can be attributed to several structural factors inherent to the recycling strategy, including the reduction in effective fiber continuity, the discontinuous nature of the cutout-based architecture, and possible limitations in the fiber–matrix interfacial adhesion, as reported by Isa et al.^21^ In addition, the presence of voids, interfacial gaps, imperfect bonding between adjacent cutouts, and localized regions deficient in either matrix or reinforcement contributed to stress concentration and crack initiation, leading to premature failure. These observations are consistent with the ultrasonic inspection results, which revealed heterogeneous consolidation regions within the laminates, in agreement with the findings reported by Song et al.^22^

The strain at break remained varied from 0.5 to 1.3%, and the ANOVA shows that the obtained value for the S13 sample is different from those determined for S21 and R21. The S21 laminate processed with square scraps presents the highest elongation at break (1.3%), and the S13 the lowest value (0.5%). In a general way, these results indicate predominantly brittle fracture behavior. Such behavior is characteristic of carbon-fiber-reinforced composites, with limited plastic deformation; however, in the present study, the observed behavior may be attributed to the presence of internal defects and nonuniform stress transfer across the discontinuous structure. In addition, the incorporation of neat PA6 between preconsolidated cutouts may have resulted in localized interfacial regions with reduced adhesion efficiency and regions rich in polymeric matrix, further contributing to unstable crack propagation under tensile loading.

The elastic modulus varied from 10.1 to 13.6 GPa. Similar to the behaviors observed for tensile strength and elongation at break, the elastic modulus of S13 laminate is statistically different from those obtained for S21 and R21 (Table 3). However, the measured values present the same order of magnitude, indicating that laminate stiffness remained primarily governed by the intrinsic rigidity and orientation of the carbon fibers, even within a discontinuous architecture.

When comparing the mechanical properties of neat PA6 (tensile strength of ∼72 MPa, elastic modulus of 3 GPa, and elongation at break of ∼11%, according to^23^ with those of the recycled laminates processed in this study, differences are observed in tensile strength, elastic modulus, and elongation at break. A significant increase in elastic modulus was achieved for all recycled laminates, with stiffness values approximately three times higher than those reported for the neat PA6. This increase demonstrates the reinforcing effect of the carbon fibers, even within a discontinuous recycled architecture.

Despite the limited improvement in tensile strength and mainly in elongation at break, this comparison shows that the recycled laminates maintained efficient stress transfer under elastic deformation, although discontinuities and interfacial defects restricted their load-bearing capability at higher strain levels. The absence of significant gains in tensile strength may be associated with the reduced effective fiber continuity, void content, imperfect bonding between adjacent cutouts, and localized stress concentration regions generated during reprocessing.

Overall, the results demonstrate that the grinding-free recycling strategy is capable of preserving an important fraction of the stiffness provided by the original composite structure, while partially compromising strength and ductility. Consequently, the recycled laminates show potential for applications with moderate mechanical requirements.

The square and rectangular cutout geometries were selected to investigate whether the directional continuity of the recycled composite affects the mechanical response of the reprocessed laminates. Square cutouts have equivalent dimensions along the two principal in-plane directions and therefore provide a geometrically balanced arrangement, whereas rectangular cutouts introduce a preferential direction associated with their longer dimension. When this longer dimension is favorably oriented relative to the loading direction, a greater continuous extent of the original composite is preserved along the load path, potentially improving stress transfer and reducing the relative influence of interfaces between adjacent cutouts. These interfaces may act as regions of stress concentration and preferential sites for damage initiation. Consequently, the results should not be interpreted as indicating that either square or rectangular cutouts are intrinsically superior. Rather, the mechanical performance depends on the interaction between cutout geometry, orientation, stacking configuration, and loading direction. This relationship is particularly relevant for recycled laminates because scrap geometry and placement can therefore be considered design variables for tailoring the mechanical response according to the intended loading condition.

An additional architectural parameter that warrants systematic investigation is the orientation of the composite cutouts relative to the loading direction. In the present experimental design, the longer dimension of the rectangular R21 cutouts was aligned with the tensile loading direction. Therefore, the present results do not allow the effect of angular orientation to be independently quantified. Alternative arrangements, including 45° and 90° orientations or mixed-angle stacking sequences, could modify the continuity of the reinforcement along the load path, the number and orientation of intercutout interfaces, and consequently the mechanisms of stress transfer and damage propagation. A systematic comparison of these configurations would therefore be valuable for separating the effects of cutout geometry from those of orientation and for tailoring the anisotropic mechanical response of recycled CF/PA6 laminates. This aspect should be systematically investigated in future studies.

The three-point flexural properties of the different laminate configurations investigated in this study are summarized in Table 3. Overall, only slight variations were observed among the obtained values, indicating that neither the number of layers nor the cutout geometry exerted a significant influence on the flexural response of the recycled composites. This behavior suggests that, under bending loading, the laminates exhibited relatively similar stress distribution and deformation mechanisms, despite differences in stacking architecture and consolidation quality.

However, a substantial reduction in flexural strength was observed when compared with recycled CF/PA6 composites processed in this work with those from the literature.^24^ According to Dong et al.,^24^ PA6 composites containing 9 and 29 wt % ground carbon fibers exhibited flexural strength values of approximately 170 MPa. The lower flexural performance observed in the composites prepared in this study can be attributed to the reduced load transfer efficiency between adjacent cutouts, the presence of voids and interfacial defects, as well as the limited continuity of the reinforcement phase throughout the laminate structure. These factors favor localized stress concentration during bending, leading to premature crack initiation and propagation.

A comparison with Santos et al.^14^ further clarifies the specific contribution of the present study. Both studies employ a grinding-free recycling approach based on the direct reprocessing of CF/PA6 composite scraps by hot compression molding; however, the experimental variables and objectives are distinct. Santos et al.^14^ primarily investigated the effect of additional PA6 content (10–40 wt %) using a random arrangement of the composite scraps, whereas the present study maintains the additional PA6 content at 15 wt % and systematically varies the geometry and stacking configuration of the recycled cutouts. At this fixed composition, tensile strength varied from 32.8 to 62.9 MPa and elastic modulus from 10.3 to 16.1 GPa, corresponding to differences of approximately 92% and 56%, respectively, between the lowest- and highest-performing configurations. This pronounced variation at constant composition and processing conditions indicates that scrap architecture itself plays an important role in governing the mechanical response of the recycled laminate. Therefore, while Santos et al.^14^ established the basis for grinding-free reprocessing and demonstrated the influence of additional matrix content, the present results extend this approach by identifying the controlled spatial organization of composite scraps as an additional design parameter for tailoring the mechanical properties of recycled CF/PA6 laminates. Absolute performance values between the two studies should nevertheless be compared cautiously because differences in matrix content, scrap architecture, and mechanical testing conditions may independently affect the measured properties.

Considering the mechanical results as a whole, the statistical analysis further supports the different effects of laminate configuration on tensile and flexural behavior. One-way ANOVA followed by Tukey’s HSD test showed that S13 exhibited significantly lower tensile strength, elastic modulus, and elongation at break than S21 and R21 (*p* < 0.05), whereas no statistically significant differences were observed between S21 and R21 for these tensile properties (*p* > 0.05). In contrast, no statistically significant differences were detected among S13, S21, and R21 for the evaluated flexural properties (*p* > 0.05), despite the numerical variations in their mean values. These statistical results indicate that, within the investigated configurations, laminate architecture had a more pronounced influence on the tensile response, whereas the flexural response was statistically comparable among the three configurations.

It should be noted that the mechanical properties reported for the original CF/PA6 composite^20^ and neat PA6 ^23^ were used as reference benchmarks rather than experimental controls. Therefore, differences relative to the recycled laminates cannot be attributed exclusively to the reprocessing cycle.

### 3.5 Fractographic Analysis

Figure 7 presents FEG-SEM images acquired at different magnifications (75×, 250×, 1000×, and 5000×) of the fracture surfaces generated during tensile testing of the laminates S13, S21, and R21, manufactured from reprocessed square and rectangular cutouts arranged in one- and two-layer configurations. At low magnification (75×), S13, processed with one layer of laminate (Figure 7a), exhibits the most homogeneous fracture surface, characterized by well-packed fiber-rich regions and localized matrix-rich areas. In contrast, S21 and R21, processed with semipregs, show more heterogeneous fracture morphologies, with detached fibers, matrix-rich regions, large voids, and intralaminar cracks, reflecting the influence of the two-layer architecture on fracture behavior (Figure 7a).

**Figure 7:**
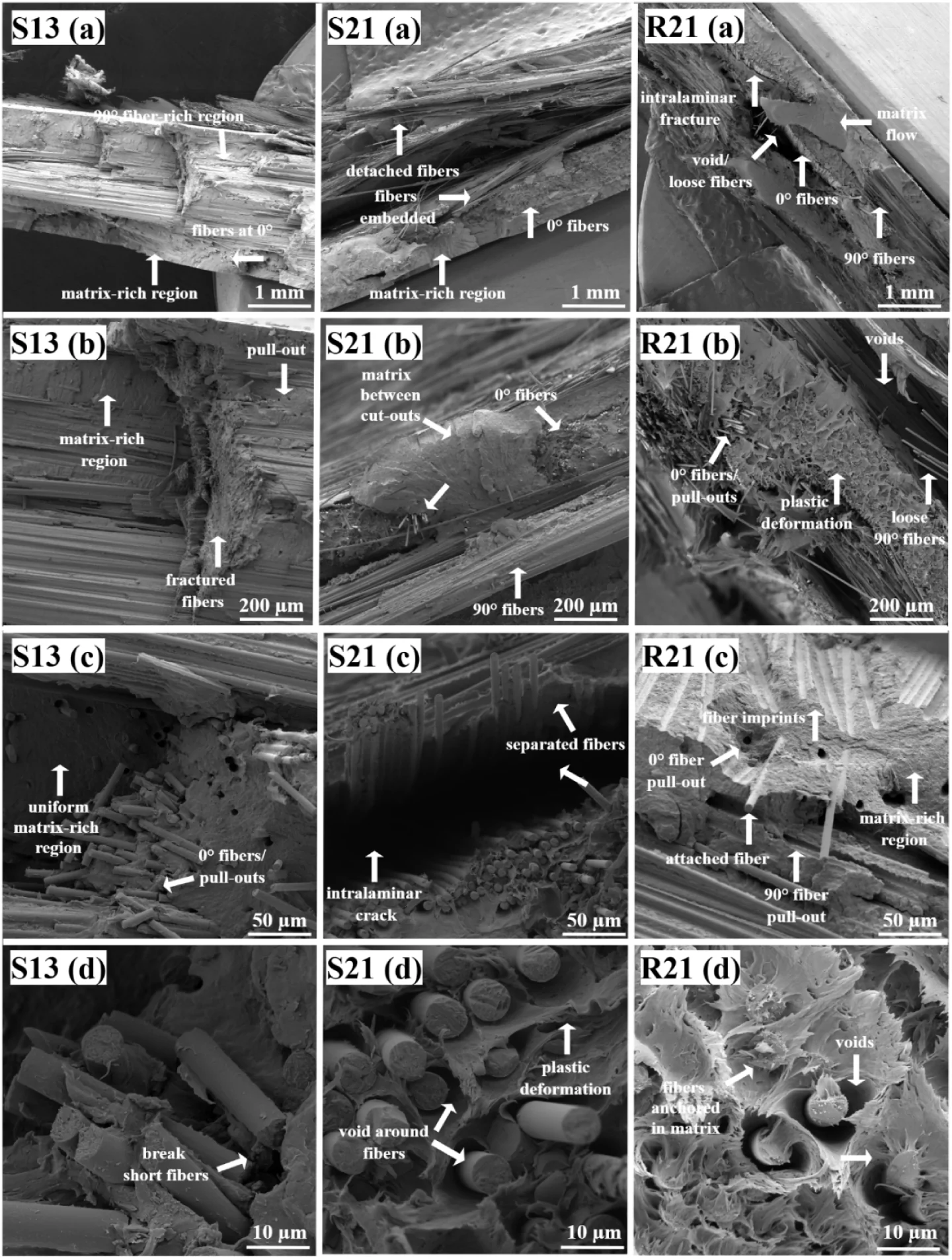
FEG-SEM images of specimens after tensile testing at magnifications of (a) 75×, (b) 250×, (c) 1000×, and (d) 5000×.

At intermediate magnifications (250× and 1000×, Figure 7b,c), S13 exhibits limited fiber pull-out and a relatively homogeneous fracture surface characterized by matrix-rich regions and short fractured fibers, likely generated during the cutout preparation process. In contrast, S21 and R21 revealed interfacial discontinuities between adjacent cutouts, fiber pull-out, voids, and intralaminar cracks. Additionally, R21 showed fiber imprints on the matrix and fibers partially embedded within the polymer, indicating localized stress transfer before failure.

At the highest magnification (5000×, Figure 7d), distinct interfacial features became more apparent. S13 predominantly exhibited short fractured fibers, whereas S21 presented voids surrounding some fibers, suggesting localized interfacial debonding. In contrast, R21 revealed fibers firmly anchored to the matrix, accompanied by matrix deformation and plastic deformation of the matrix. These features indicate effective stress transfer at the fiber-matrix interface and are commonly associated with strong interfacial adhesion.

Overall, the fractographic analysis demonstrates that cutout geometry and stacking architecture influenced the fracture mechanisms, fiber distribution, and failure behavior of the reprocessed laminates. Although S13 exhibited the most homogeneous microstructure, the more complex fracture features observed in S21 and R21 are consistent with their superior tensile performance, suggesting that enhanced interfacial interactions contributed to improved load transfer and mechanical response.

A comparison of fractographic observations with the acid digestion and ultrasonic inspection results reveals a good correlation among the different characterization techniques. Laminate S13 exhibited the most homogeneous microstructure, consistent with the lower void content determined by both acid digestion and ultrasonic analysis. In contrast, the two-layer laminates, S21 and R21, showed a higher prevalence of voids, as evidenced by FEG-SEM images, ultrasonic maps, and the higher void fractions quantified through acid digestion.

The mechanical performance was also closely associated with the observed fracture characteristics. As shown in Table 3, laminate S13 exhibited lower ultimate tensile strength and elongation at break than the other laminates, despite its relatively homogeneous fracture surface and the absence of pronounced matrix deformation or fiber pull-out. Although S13 exhibited an apparently more homogeneous microstructure, the interfaces between adjacent cutouts may represent discontinuities in the reinforcement load path, with PA6-rich interfacial regions potentially limiting stress transfer between neighboring composite pieces. Similar matrix-rich regions at scrap joints have been reported for reprocessed CF/PA6 laminates.^15^ Therefore, the lower tensile performance of S13 may be associated with stress concentration and less efficient load transfer across these intercutout regions rather than with microstructural homogeneity alone. Conversely, S21 and R21 displayed comparable tensile properties and fracture surfaces characterized by extensive matrix deformation, fiber pull-out, cusp formation, intralaminar cracking, and the presence of loose fibers. Such features have been reported for CF/PA6 composites^25^ and are consistent with more effective energy-dissipation and load-transfer mechanisms, contributing to the improved mechanical response of these laminates.

Overall, the combined microstructural, nondestructive, and mechanical characterization results indicate that the S21 and R21 configurations exhibited the most favorable balance between structural integrity and mechanical performance among the configurations investigated. However, because layer number and individual layer thickness vary simultaneously between S13 and the two-layer configurations, the improved performance cannot be attributed exclusively to the number of layers.

The strong agreement among the different characterization techniques confirms the reliability of the observed trends and highlights the critical role of laminate architecture in governing damage development and failure mechanisms. In particular, the superior performance observed for S21 and R21 demonstrates that laminate architecture is strongly associated with the resulting mechanical response, although the individual contributions of layer number and layer thickness cannot be fully decoupled within the present experimental design. These findings contribute to the understanding of the structure–property relationships in recycled carbon-fiber thermoplastic composites and support the feasibility of new studies to improve this recycling approach in applications with reduced structural requirements, but which meet the final usage requirements.

## 4 Conclusion

In this work, a grinding-free recycling strategy for CF/PA6 composite waste was successfully developed through the controlled rearrangement of composite cutouts followed by hot compression molding. The proposed approach enabled the recycling of CF/PA6 laminate residues, preserving part of the original fiber architecture and avoiding additional fiber degradation associated with conventional grinding-based recycling routes. The results demonstrated that cutout geometry and stacking configuration strongly affected laminate consolidation, void content, crystallization behavior, and mechanical performance. Ultrasonic inspection and acid digestion showed a strong correlation regarding the identification of poorly consolidated regions and internal voids. Among the investigated configurations, laminate S13 exhibited the lowest void content and the most homogeneous consolidation, whereas multilayer laminates presented higher defect concentrations associated with cutout displacement and interfacial discontinuities during processing.

DSC analyses indicated that the recycling process preserved the thermal characteristics of PA6, while some recycled laminates exhibited increased crystallinity compared with the original CF/PA6 scraps, likely due to the nucleating effect of carbon fibers combined with the thermal history imposed during reprocessing. The recycled laminates containing 85 wt % CF/PA6 production waste and 15 wt % virgin PA6 exhibited tensile strengths ranging from 32.8 to 62.9 MPa and elastic moduli from 10.3 to 16.1 GPa, depending on the cutout configuration. The difference between the lowest- and highest-performing configurations corresponded to approximately 92% in tensile strength and 56% in elastic modulus, demonstrating that cutout arrangement is a key parameter governing the mechanical response of the recycled laminates. Although the mechanical properties remained below those of the original composite, the results demonstrate that mechanically functional consolidated laminates can be produced with 85 wt % recycled composite content and a limited addition of virgin PA6. The combined results therefore establish a direct relationship between cutout configuration, consolidation, and mechanical performance, while demonstrating the feasibility of retaining a high fraction of CF/PA6 production waste in a new material. Among the recycled configurations, the laminate S21 resulted in the best tensile performance.

Fractographic analysis showed that the laminate S13 exhibited a relatively homogeneous fracture morphology, whereas S21 and R21 displayed more complex fracture features, including matrix deformation, fiber pull-out, and intralaminar cracking. These fractographic aspects are indicative of enhanced energy absorption and more effective stress redistribution during loading, which is consistent with the superior tensile performance observed for the two-layer configurations.

Overall, the proposed recycling strategy represents a simple and potentially scalable approach for the valorization of CF/PA6 manufacturing scraps, making them attractive candidates for applications without structural requirements. These findings contribute to the advancement of sustainable recycling technologies for thermoplastic composites and provide valuable insights into the structure–property relationships that govern the performance of reprocessed continuous-fiber composite materials. Nevertheless, the effect of cutout orientation relative to the loading direction was not independently investigated in the present experimental design. Future studies incorporating different angular arrangements, such as 45° and 90° orientations, could provide further insight into the anisotropic mechanical response and contribute to the optimization of scrap architectures for specific loading conditions.
